# Supplementary figures and images for: PLCγ2 deficiency compromises systemic immune tolerance and erodes myelin homeostasis while enhancing oxidative metabolism in the mouse brain
Source: bioRxiv. 2026 Jul 14:2026.07.13.738356. Preprint. [Version 1] doi: 10.64898/2026.07.13.738356 (PMC13405013; doi:10.64898/2026.07.13.738356)

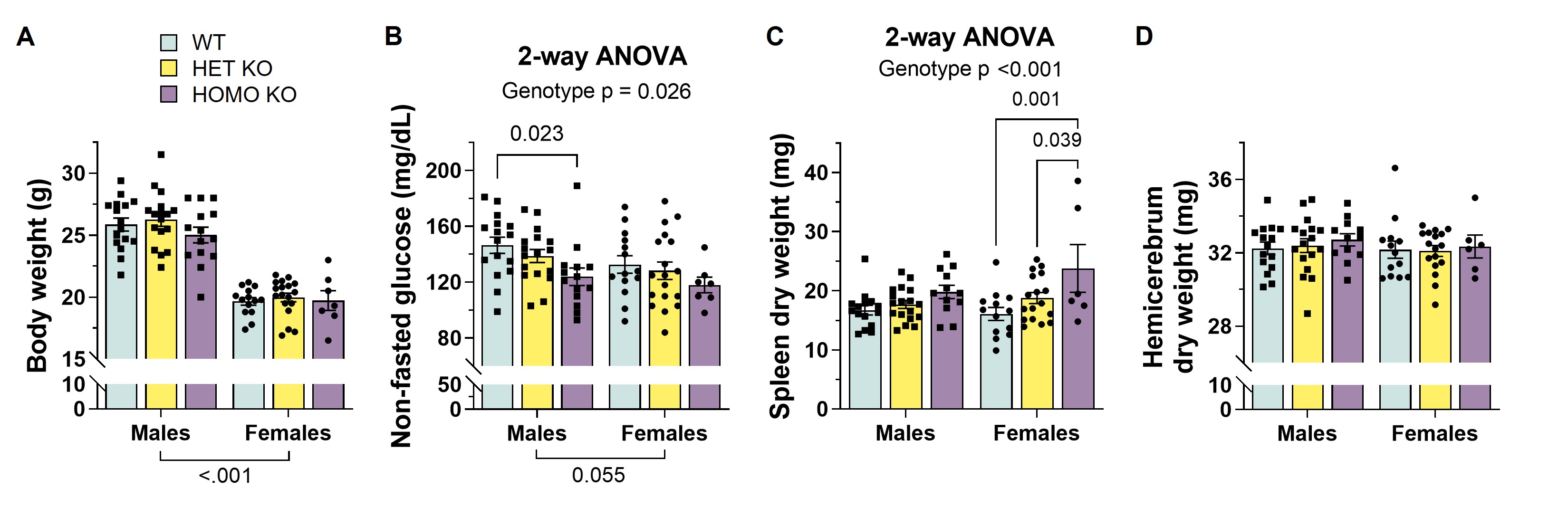

Supplement: Supplement 1 — Figure S1. Sex-stratified body weight, blood glucose, spleen weight, and brain weight in Plcg2-deficient mice, including an independent cohort. (A) Body weight in male and female Plcg2 WT, Het KO, and Homo KO littermates at the 3-month endpoint, combining untreated animals (Figure 1) and an independent PBS-treated control cohort. (B) Non-fasted blood glucose at the 3-month endpoint in male and female Plcg2 littermates of each genotype from both cohorts. (C) Dry spleen weight at the 3-month endpoint in male and female Plcg2 littermates of each genotype from both cohorts. (D) Hemicerebrum dry weight at the 3-month endpoint in male and female Plcg2 littermates of each genotype from both cohorts. Data are presented as mean ± SEM; individual points represent individual animals (males, squares; females, circles). Two-way ANOVA with factors genotype and sex was followed by Tukey’s post hoc tests assessing simple genotype effects within each sex; p values (adjusted for multiple comparisons) < 0.1 are indicated in the graphs (A–D). [file media-1.jpg]

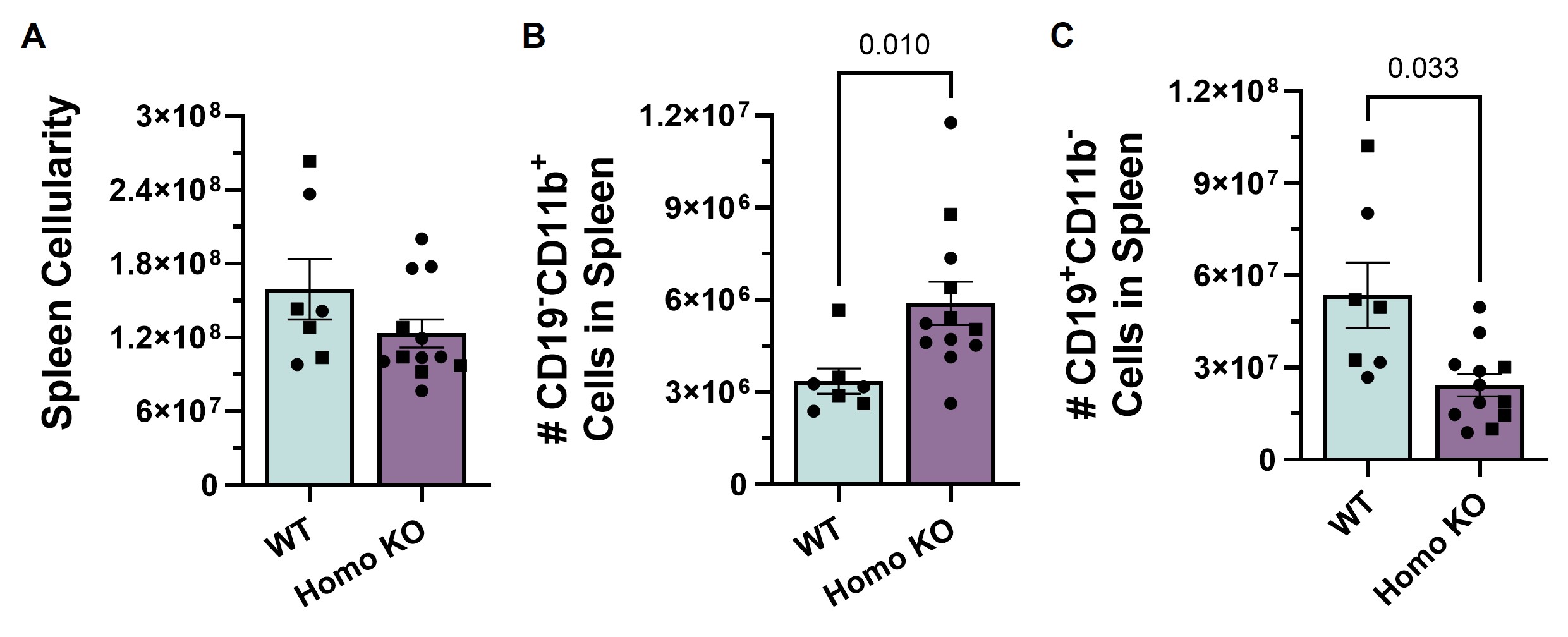

Supplement: Supplement 2 — Figure S2. Absolute spleen cellularity and myeloid/B-cell counts in Plcg2-deficient mice. (A) Total live splenocyte counts in WT and Plcg2 Homo KO mice. (B) Absolute number of CD19−CD11b+ myeloid/innate-enriched cells per spleen. (C) Absolute number of CD19+CD11b− B cells per spleen. Data correspond to the same animals shown in Figure 2 and are presented as mean ± SEM; individual points represent individual animals (males, squares; females, circles). Statistical significance was assessed using unpaired two-tailed t tests when data passed normality and homoscedasticity tests (A), Welch’s t test when F tests indicated unequal variances between groups (C), or Mann–Whitney tests when data did not meet normality assumptions (B); p values < 0.1 are indicated in the graphs. [file media-2.jpg]

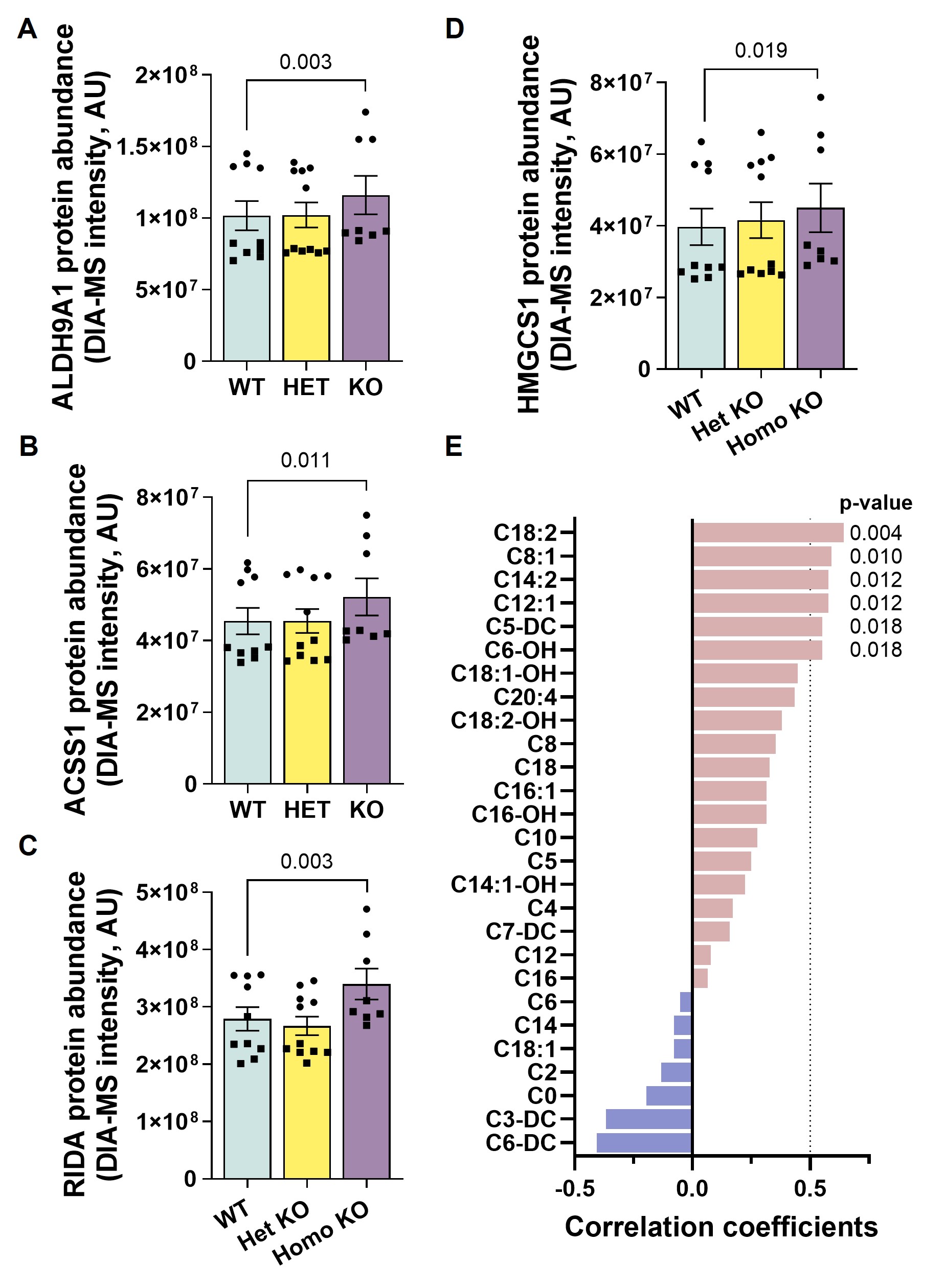

Supplement: Supplement 3 — Figure S3. Extended analyses of mitochondrial lipid and amino-acid catabolism in Plcg2-deficient brains. (A-D) DIA-MS proteomics was used to quantify additional mitochondrial matrix enzymes, including ALDH9A1, ACSS1, RIDA, and HMGCS1, in cerebrum from Plcg2 WT, Het KO, and Homo KO mice, with normalized protein abundances shown for each genotype. (E) Targeted LC/MS acylcarnitine metabolomics was used to assess correlations between individual acylcarnitine species and Plcg2 genotype; bar plots display correlation coefficients and corresponding p-values for selected short-, medium-, and long-chain acylcarnitines. Data are presented as bar plots with each point representing an individual animal; statistical models and multiple-testing corrections used for proteomic and metabolomic analyses are described in the Methods. [file media-3.jpg]
